# Supplementary material for: The Small Heat Shock Protein, HSPB1, Interacts with and Modulates the Physical Structure of Membranes
Source: Int J Mol Sci. 2022 Jun 30;23(13):7317. doi: 10.3390/ijms23137317 (PMC9266964; doi:10.3390/ijms23137317)
Supplement: Supplementary file 1 [file ijms-23-07317-s001.zip › ijms-1772002-supplementary.pdf]

Supporting Information

The small heat shock protein, HSPB1, interacts with and modulates the physical structure of membranes

Balint Csoboz[1,2*], Imre Gombos[1*], Zoltán Kóta[3,4], Barbara Dukic[1], Éva Klement[1,4],Vanda Varga-Zsíros[1], Zoltán Lipinszki[1],Tibor Páli[3], László Vigh[1], Zsolt Török[1]

1 Institute of Biochemistry, Biological Research Centre, Szeged, Hungary;

2 Institute of Medical Biology, University of Tromsø, Tromsø, Norway;

3 Institute of Biophysics, Biological Research Centre, Szeged, Hungary,

4 Single Cell Omics Advanced Core Facility, Hungarian Centre of Excellence for Molecular Medicine, Szeged Hungary

[*]shared first authorship

Table of contents:

Figure S1. Distribution of HSPB1 and raft associated proteins in the membrane fractions after heat shock.

Figure S2. HSPB1 protein accumulates in the membrane after heat shock.

Figure S3. A) HSPB1 expression in *E. coli* cells upon IPTG induction.

Figure S3. B) HSPB1 expression in B16F10 and B16-F10/HSPB1 cells.

Materials and Methods

References

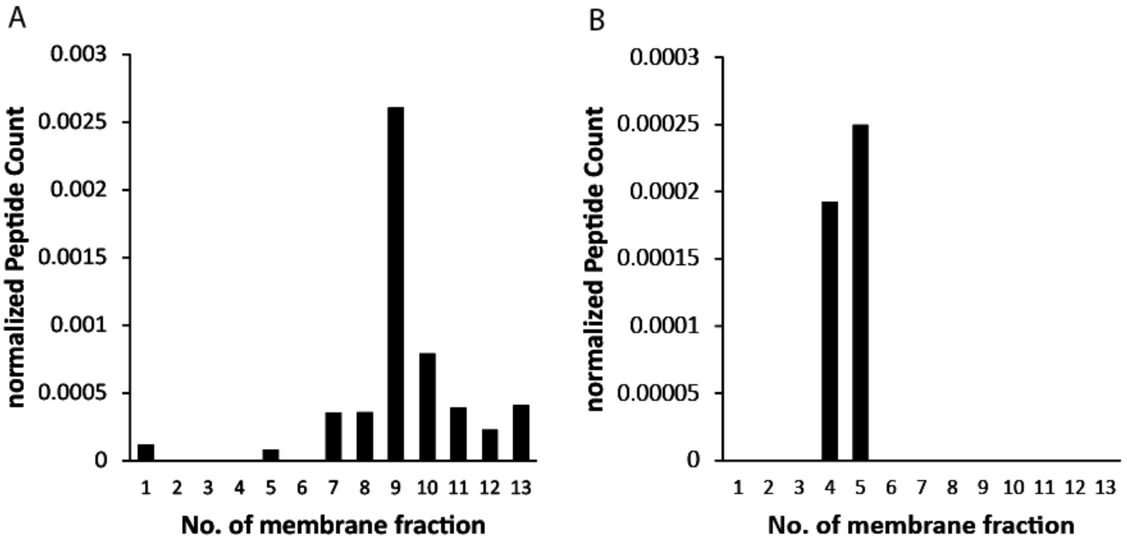

Figure S1. Distribution of HSPB1 and raft associated proteins in the membrane fractions after heat shock. B16-F10 cells were subjected to heat shock at 42 °C for one hour, then incubated for 24 hours at 37 °C. After the recovery period, subcellular isolation of membranes and rafts was carried out. Fraction from 1-13 represent different membrane regions collected for mass spectrometry analysis. Earlier fractions correspond to cholesterol rich membrane regions. The figure shows the peptide count for **(A)** HSPB1 and **(B)** the typical raft associated protein Flotillin-1 in each fraction.

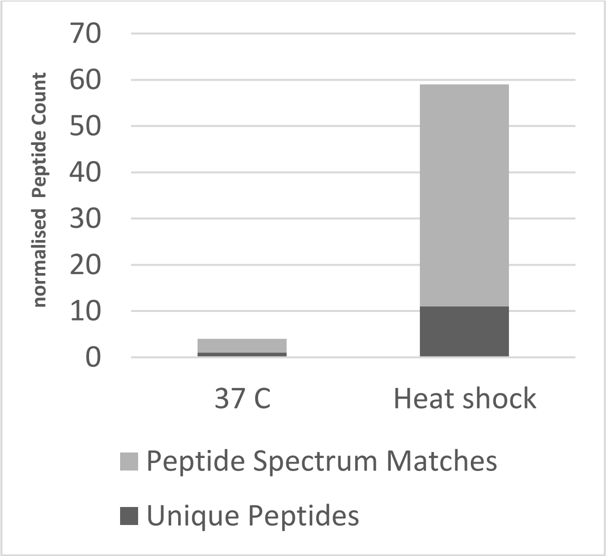

Figure S2. HSPB1 protein accumulates in the membrane after heat shock. B16-F10 cells were subjected to heat shock at 42 °C for one hour or left untreated, followed by an incubation for 24 hours at 37 °C. After the recovery period, the subcellular isolation of membranes and rafts was carried out. The figure shows the cumulative peptide counts in all membrane fractions for HSPB1 between cells kept at 37 °C and heat treated at 42 °C.

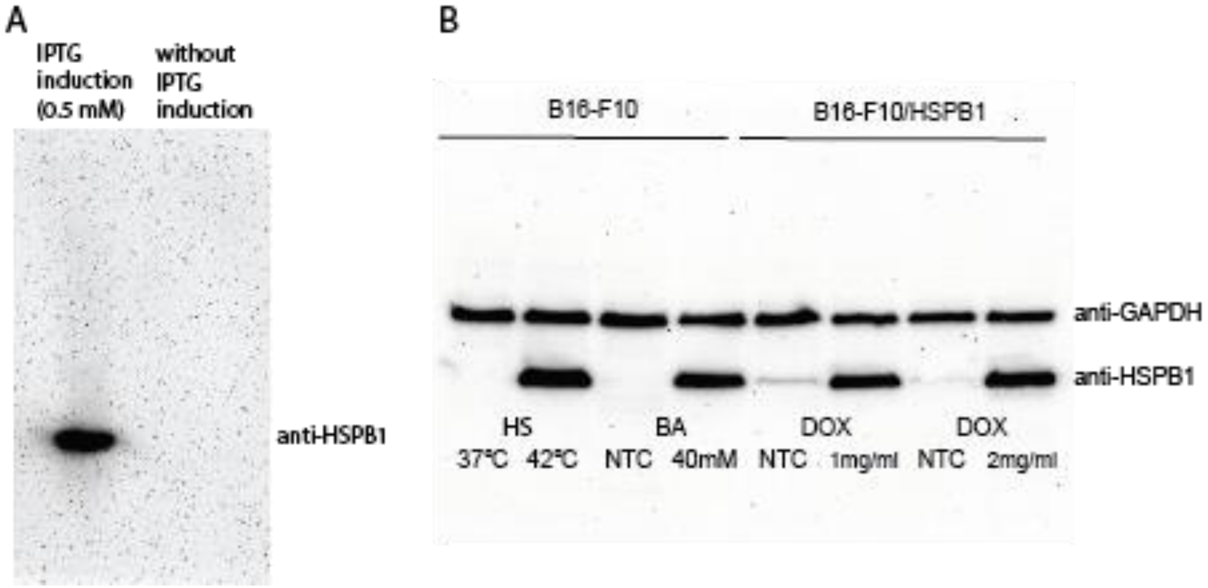

Figure S3. (A) HSPB1 expression in *E. coli* cells upon IPTG induction. Recombinant human HSPB1 was expressed in *Escherichia coli* BL21 (DE3) cells by using the pAK3038Hsp27 plasmid. E. coli cells harboring the pAK3038Hsp27 plasmid were induced with isopropylthiogalactoside (IPTG) at a final concentration of 0.5 mM. Cells were incubated for 3 h upon IPTG induction, then harvested and lysed. The expression of HSPB1 was assessed by western blotting. **(B) HSPB1 expression in B16F10 and B16-F10/ HSPB1 cells.** B16-F10 cells were subjected to heat shock (HS) at 42 °C or to benzyl alcohol (BA) at 40mM for one hour, cells were harvested for analysis after 24 h at 37 °C. In B16-F10/ HSPB1 the expression of HSPB1 was induced by the addition of doxycycline hyclate (DOX) to the cell culture media (1 or 2 μg/ml) for 24 h before the experiments. NTC refers to non-treated control. The expression of HSPB1 was assessed by western blotting.

Materials and Methods

Membrane fractionation

The preparation of membrane fractions was executed as described inref 1. Briefly, B16-F10 cells were heat shocked at 42 °C for one hour or left untreated. For heat shock treatments, the plates were immersed in a water bath set to the indicated temperature (±0.1°C). The cells were allowed to recover for 24 hours. After the recovery period, the subcellular isolation of membranes and rafts was carried out as follows. Fractions were isolated by washing the cells with ice-cold PBS, collecting by scraping, lysing with the addition of glass beads (0.9–2 mm) (Sigma Aldrich), and centrifuged at 1000 rpm for 5 minutes. The cell lysate was diluted with 80 % sucrose (Sigma Aldrich) and 4 ml was put into an Ultra Clear SW41 rotor tube (Beckman Coulter, Brea CA, United States). Four ml of 30 % sucrose was layered on the sample on which another 4 ml of 5 % sucrose was layered. The samples were centrifuged (SW41 rotor, 35 000 rpm, 22 h, 2 °C) (Beckman Coulter, Brea CA, United States). Subsequently, 1 ml fractions were collected and proteins were precipitated with TCA/acetone (Sigma Aldrich) for proteomics.

Mass spectrometry-based proteomics of membrane fractions

Protein pellets from each fraction were denatured in 6 M GuHCl. Disulfide bridges were reduced with DTT (Sigma Aldrich), then free sulfhydryls were alkylated with iodoacetamide (Sigma Aldrich). To the diluted samples, trypsin was added and incubated at 37 °C overnight. After digestion, the samples were acidified and desalted. The dried samples were reconstituted in 0.1% formic acid (Sigma Aldrich) and analyzed by LC-MS/MS on an Orbitrap Fusion Lumos mass spectrometer (Thermo Fischer Scientific) coupled on-line to a Waters (Perth, Australia) nanoAcquity UPLC in data dependent acquisition mode applying higher-energy collisional dissociation (HCD) as follows. The sample was loaded onto a Waters Symmetry trap column (C18, 5 μm, 100 Å 180 mm x 20 mm) in 99% solvent A (0.1% formic acid in water) at a flow rate of 5 μl/min for 3 min, then fractionated on a Waters BEH column (C18, 1.7 μm, 130 Å, 75 μm x 250 mm) by increasing solvent B (0.1% formic acid in acetonitrile) from 10% to 40% in 30 min at a flow rate of 300 nl/min. MS survey scans (m/z 380–1580; AGC 400,000; max inject time 50 ms, resolution 60,000, RF 30) were followed by HCD scans (m/z auto; AGC 50,000; max inject time 22 ms, resolution 15,000; isolation width 1.2 Da; cycle time 3 s, normalized collision energy 35%) on the most abundant multiply charged ions with a minimum intensity threshold of 50,000, then excluded for 15 s. Raw data were converted into peak lists using Proteome Discoverer (v 1.4) (Thermo Fischer Scientific). Database search was performed by Protein Prospector (v 5.22.0) against mouse entries in the UniProtKB.2017.9.19 database concatenated with randomized sequences and also considering general contaminants (83946 entries). A precursor mass tolerance of 5 ppm and a fragment mass tolerance of 20 ppm was used. Only fully tryptic peptides were considered with a maximum of two missed cleavages. Carbamidomethylation of Cys residues was used as fixed modification and Met oxidation, acetylation of protein N-termini, and pyroglutamic acid formation of peptide N-terminal Gln residues as variable modifications. Protein and peptide hits were accepted with FDR<1%. Relative quantitation was performed using spectral counting on the merged database search results of the respective fractions.

Stable expression of HSPB1 in B16F10 cells

The HSPB1 cDNA was purchased from Origene (Rockville, Maryland, USA) and was cloned into the pcDNA 4/TO vector (Thermo Fischer Scientific). For inducible protein expression, pcDNA6/TR (Thermo Fischer Scientific) was used as a source of tetracycline repressor. B16F10 cells were transfected by using ExGen 500 (Thermo Fischer Scientific), according to the manufacturer's instructions, with pcDNA4/TO-HSPB1 and pcDNA6/TR to achieve inducible protein expression. To create single cell clones the transfected cells were selected by the addition of blasticidine and zeocin (Thermo Fischer Scientific). The expression of HSPB1 was induced by the addition of doxycycline hyclate (Sigma Aldrich, D9891) to the cell culture media for 24 h (2 μg/ml).

Western blotting

The expression induction of HSPB1 in *E. coli* BL21 (DE3) (Thermo Fischer Scientific) containing the pAK3038Hsp27 plasmid [2] and in B16-F10/HSPB1 cells was assessed by western blot. For inducible protein expression the *E. coli* cells were induced with isopropylthiogalactoside (IPTG) at a final concentration of 0.5 mM. Cells were incubated for 3 hours upon IPTG induction, then harvested. B16-F10 (ATCC) cells were subjected to heat shock at 42 °C or to benzyl alcohol (BA) at 40 mM for one hour, cells were harvested for analysis after 24 hours. B16-F10 cells treated with heat shock or with BA were included in the experiments to compare the artificially induced levels of HSPB1 to natural HSBP1 inducers. In B16-F10/HSPB1 the expression of HSPB1 was induced by the addition of doxycycline hyclate (DOX) to the cell culture media (1 or 2 μg/ml) for 24 hours before the experiments, cells were harvested for analysis after 24 hours.

Escherichia coli, B16-F10, and B16-F10/HSPB1 cells were lysed in Laemmli buffer and an equal amount of proteins were run on SDS/PAGE and transferred to a PVDF membrane. Membranes were probed with anti-GAPDH (Sigma Aldrich, G8795) and/or anti-HSPB1 (SMC-161, StressMarq) antibodies. Signals were visualized using HRP-conjugated secondary antibodies.

As Figure S3B shows the HSPB1 levels induced upon the 2 mg/ml doxycycline treatment were comparable to the ones induced by heat shock or BA treatment. Therefore, this concentration was used for further experiments.

References

1. Csoboz, B. *et al.* Chemotherapy induced PRL3 expression promotes cancer growth via plasma membrane remodeling and specific alterations of caveolae-associated signaling. *Cell Commun. Signal.* **16**, 51 (2018).

2. Jakob, U., Gaestel, M., Engel, K. & Buchner, J. Small heat shock proteins are molecular chaperones. *J. Biol. Chem.* **268**, 1517–20 (1993).
